# Geomorphic effects of recurrent outburst superfloods in the Yigong River on the southeastern margin of Tibet

**DOI:** 10.1038/s41598-021-95194-1

**Published:** 2021-08-02

**Authors:** Kaiheng Hu, Chaohua Wu, Li Wei, Xiaopeng Zhang, Qiyuan Zhang, Weiming Liu, Brian J. Yanites

**Affiliations:** 1 Key Laboratory of Mountain Hazards and Earth Surface Processes, Chinese Academy of Sciences, Chengdu, 610041 China; 2 Institute of Mountain Hazards and Environment, Chinese Academy of Sciences, Chengdu, 610041 China; 3 Department of Earth and Atmospheric Sciences, Indiana University Bloomington, Bloomington, IN 47405 USA

**Keywords:** Hydrology, Natural hazards, Solid Earth sciences

## Abstract

Landslide dam outburst floods have a significant impact on landform evolution in high mountainous areas. Historic landslide dams on the Yigong River, southeastern Tibet, generated two outburst superfloods > 10^5^ m^3^/s in 1902 and 2000 AD. One of the slackwater deposits, which was newly found immediately downstream of the historic dams, has been dated to 7 ka BP. The one-dimensional backwater stepwise method gives an estimate of 225,000 m^3^/s for the peak flow related to the paleo-stage indicator of 7 ka BP. The recurrence of at least three large landslide dam impoundments and super-outburst floods at the exit of Yigong Lake during the Holocene greatly changed the morphology of the Yigong River. More than 0.26 billion m^3^ of sediment has been aggraded in the dammed lake while the landslide sediment doubles the channel slope behind the dam. Repeated landslide damming may be a persistent source of outburst floods and impede the upstream migration of river knickpoints in the southeastern margin of Tibet.

## Introduction

Large landslide or glacier dams and associated outburst floods have a profound impact on alpine landscape evolution around the Tibetan Plateau^1–5^. These outburst floods have double effects. On the one hand, natural long-lived dams as stable knickpoints protect upstream channels from river incision and hence retard headward erosion into the plateau interior. On the other hand, dammed lake outburst floods may control long-term valley evolution such as erosion of bedrock canyons in the Himalayas. Among these floods, so-called superfloods play a key role in bedrock plucking, boulder mobilization, and coarse grain comminution that are processes in which moderate flows have poor competence^6–9^. However, the frequency of superflood events is unusually low and few direct observations are made. Most information on the superflooding process comes from sedimentary records of outburst flood deposits downstream of breached dams^10,11^. Records of modern and ancient superflood events in a river are useful for verifying the flood geomorphic effects and hydraulic hypotheses.

**Figure 1:**
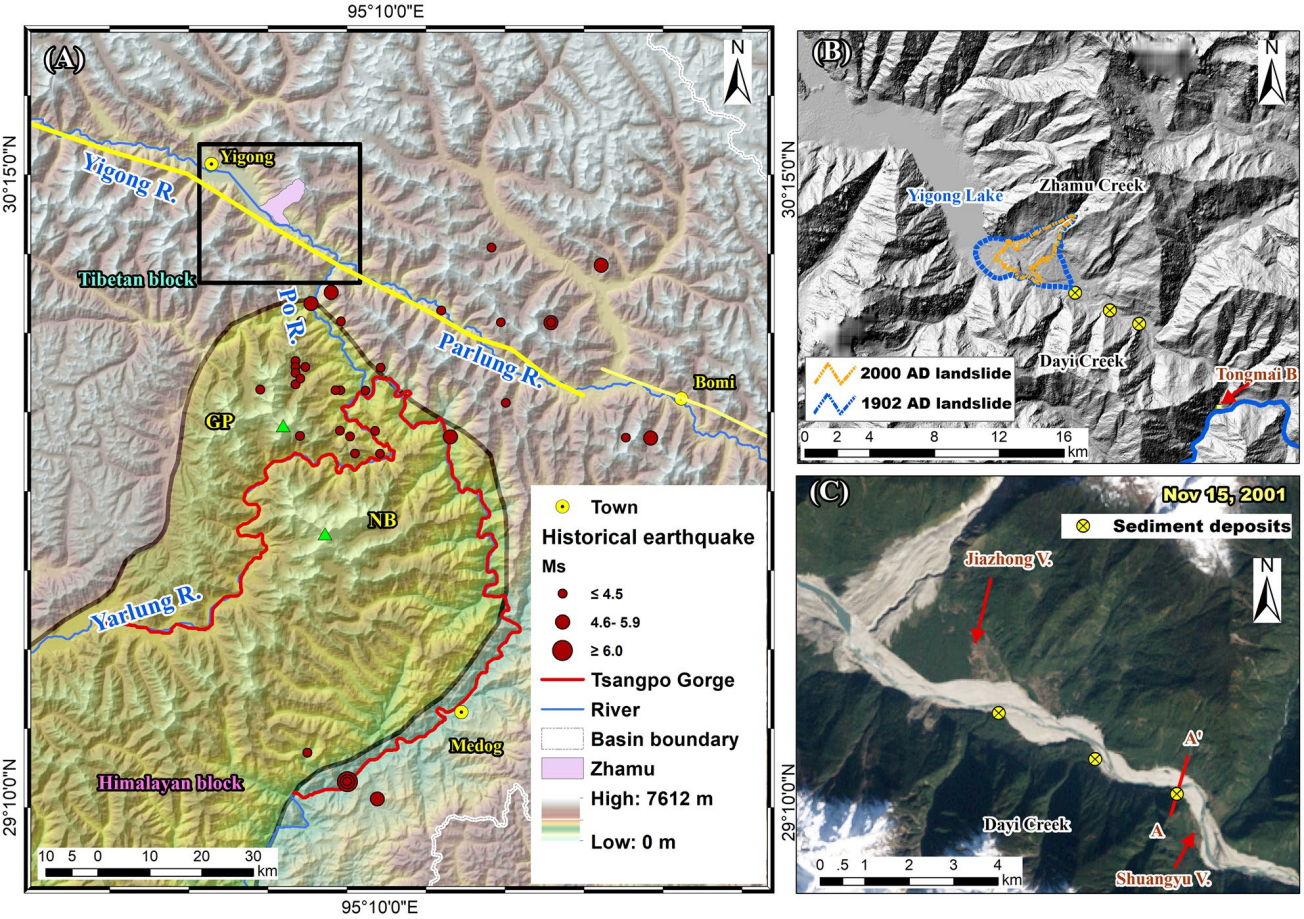
(**A**) Location of the Yigong River and the Yarlung Tsangpo Gorge. The eastward flowing Yigong River turns southward and flows into the gorge after confluence with the westward flowing Parlung River at Tongmai. The topographic map was extracted from NASA’s 90 m Shuttle Radar Topography Mission elevation data. The grey line is the Yarlung Tsangpo suture zone. The yellow lines are the Jiali fault. The black box is the extension of subfigure (**B**). NB denotes the Namche Barwa massif (7782 m), and GP denotes the Gyala Peri massif (7294 m). (**B**) Topographic relief of Yigong’s downstream area mapped with the Advanced Land Observing Satellite-1 (ALOS) 12.5 m DEM. The blue and orange outlines denote the ranges of the 1902 AD and 2000 AD landslide dams, respectively. The yellow circles with black crosses denote the location of the sediment records. Tongmai Bridge is in a bedrock reach at the confluence of the Yigong and Parlung Rivers. (**C**) ETM + image on November 15, 2001. The 2000 AD flood greatly changed the river channel morphology and local topography. A–A’ marks the location of cross-sections in Fig. 2. The maps were created using a licensed ArcGIS 9.3 software (https://support.esri.com/en/downloads).

The Yigong River, a tributary of the Yarlung Tsangpo River, southeastern Tibetan Plateau, experienced two well-documented modern superfloods > 10^5^ m^3^/s in 1902 and 2000 AD^12–15^ (Fig. 1). Both events were caused by the failure of landslide dams at the same location, the Zhamu Creek confluence (Fig. 1B). In this study, we report the findings of ancient lacustrine and slack water deposits (SWDs) downstream of the Zhamu landslide during a recent field survey (Fig. 1C). The age of the deposits is estimated with radiocarbon and Optically Stimulated Luminescence (OSL) dating methods. We calculate the paleoflood peak discharge related to these deposits by using the 1-D step-backwater method and discuss the impact of the three flood events on the Yigong River’s evolution. Such direct observations and records of repeated outburst floods in the same river reach are very valuable in verifying paleoflood hydraulic reconstruction and better understanding the role of landslide dam outburst floods in the landscape evolution of the Tibetan Plateau’s eastern margin.

## Study area

The study area is downstream of the Yigong River, a tributary of the Parlung Tsangpo that flows into the Yarlung Tsangpo River through the Grant Tsangpo Gorge on the southeastern margin of the Tibetan Plateau (Fig. 1). It is surrounded by numerous summits > 6000 m a.s.l. in the Nyaiqentanglha range to the north and the Himalayan range to the south. Local topographic relief reaches up to 6 km in the gorge between the Namche Barwa (7782 m) and Gyala Peri (7294 m) massifs. The warm and humid Indian monsoon reaches further upstream of the Yigong River through the deep gorge. The average annual rainfall descends from ~ 3000 mm at the town of Medog to ~ 1100 mm at the town of Yigong. Abundant precipitation and high relief make it the most active region for monsoonal temperate glaciers with a total area of 2490 km^2^ in China.

The Yigong region is in a highly tectonically active mountain belt where the rock uplift rate is as high as 10 mm/year. The eastern syntaxis of the Himalaya, a northeastern protrusion between the Indian and Asian plates, is ~ 30 km to the south. The Jiali fault, a large WNW-ESE-trending strike-slip fault, extends from the Yigong to the Parlung Tsangpo, traversing the southeastern Tibetan Plateau. Due to active tectonic movement and high relief, frequent strong earthquakes have occurred and triggered numerous geological hazards, e.g., the Mw 8.6 Great Assam earthquake in 1950, and the Ms 6.9 Milin earthquake in 2017. The Yarlung Tsangpo suture zone is a distinct boundary between Tibetan and Himalayan source rocks (Fig. 1A). The Tibetan source area completely contains the study area. The primary outcropping rocks in the area are Proterozoic gneiss, pre-Carboniferous schist, and Carboniferous slate and sandstone. Widespread Quaternary deposits, including laterofrontal moraines, debris fans, and flood deposits, are distributed along the river valley.

The Yigong River has a drainage area of 13,500 km^2^ and a mean annual discharge of 378 m^3^/s. Two large-magnitude landslides took place in 1902 and 2000 AD at Zhamu Creek next to the exit of Yigong Lake (some English papers report the 1902 AD event having occurred in 1900) (Fig. 1). Delaney & Evans^15^ reviewed the published literature on the 2000 event and presented a reliable estimate of 115 Mm^3^ for the landslide volume in 2000. Descriptions of the 1902 event vary because first-hand data are not available, and most records in Chinese literature originate from interviews with local witnesses in 1960s. Lu et al.^16^ calculated that the deposition area was 11.6 km^2^ and the final deposition volume was 513 Mm^3^ from a 1:100,000 topographical map that was measured via an airborne survey in 1968. Zhou et al.^17^ gave a higher estimate of 1000 Mm^3^ for the 1902 landslide volume. The break of the landslide dams led to two documented modern superfloods > 10^5^ m^3^/s in the Yigong River. The floods entered the Parlung River at the Tongmai junction and then travelled through Tsangpo Gorge on the east side of the Namche Barwa and Gyala Peri peaks (Fig. 1A). Neither of the two landslide dams were completely breached, and it is argued that broad Yigong Lake resulted from the 1902 damming^15^.

## Paleoflood deposits and peak flow reconstruction

### Paleoflood deposits

**Figure 2:**
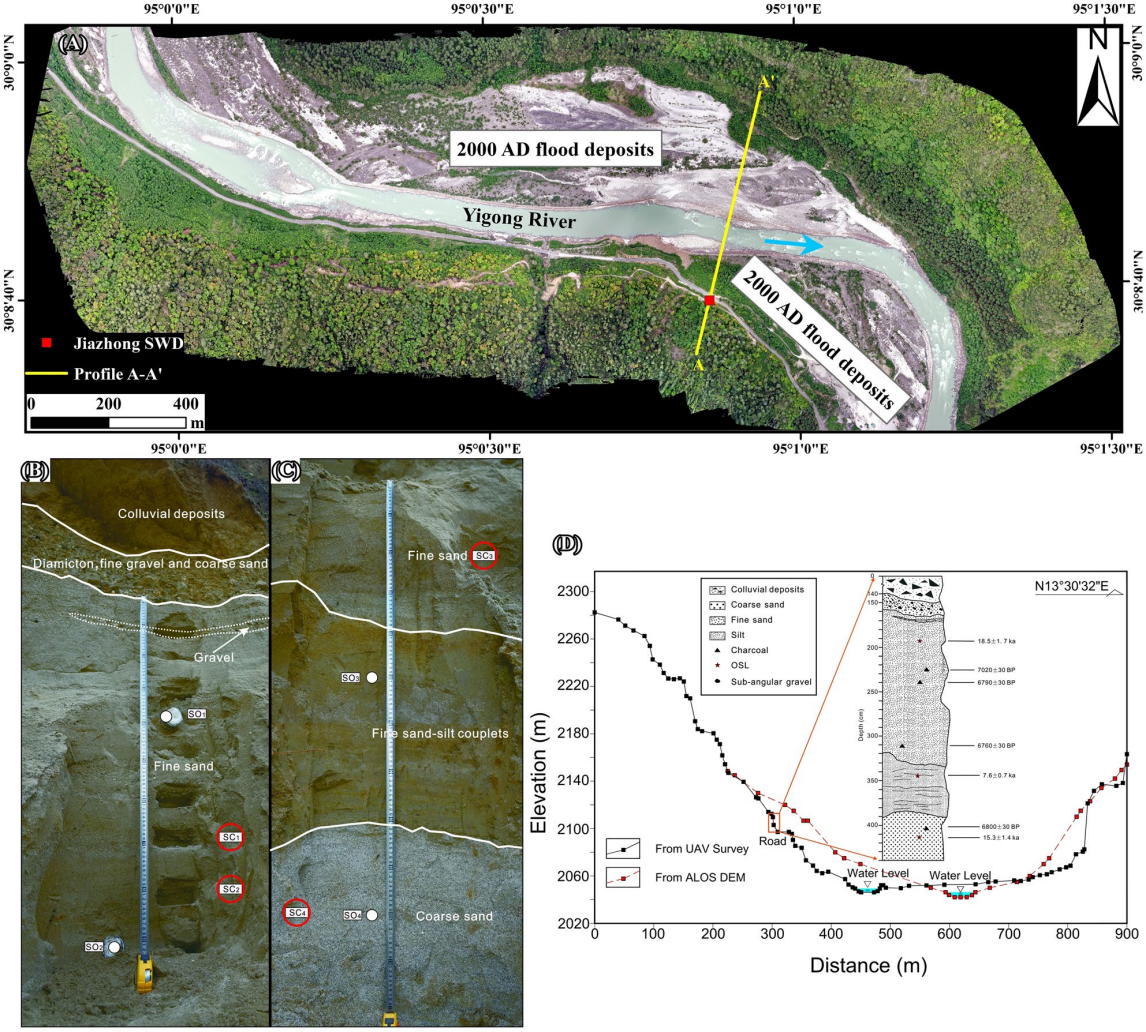
(**A**) Location of slackwater deposits (SWDs) at Shuangyu Village. Orthographic image taken on May 11, 2020, by the Dajiang unmanned aerial vehicle (UAV); (**B**) upper part of sedimentary section; (**C**) lower part of sedimentary section. The total thickness of the section is 2.6 m. SO1 to SO4 denotes luminescence dating samples and SC1 to SC4 denotes radiocarbon dating samples; (**D**) cross-section and sedimentary sequence of the Shuangyu SWD. The two cross-sections were derived from the UAV survey on May 11, 2020 (solid line) and the Advanced Land Observing Satellite (ALOS) 12.5 DEM (dashed line). The water level is estimated as ca. 3 m based on the mean discharge of 520 m^3^/s in May. The ALOS DEM is consistent with the UAV survey at the main channel and floodplain without big alteration by the 2000 AD flood, but differs by up to 25 m on riverbanks and hillslopes.

SWDs are fine-grained sediments carried in suspension in high-energy floods that are deposited in areas of low velocity, such as embayments, alcoves, and tributary junctions, and are commonly used as paleo-stage indicators (PSIs)^18,19^. One set of SWDs (N 30° 8′ 38.67″, E 95° 0′ 51.15″) was found leeward of a river bend 8.3 km downstream of the 2000 AD dam near Shuangyu village (Figs. 1C, 2A). This SWD is 52 m above present river level and overlain by a 1.4 m thick landslide deposit. The sequence is nearly horizontally bedded and comprises four units (Fig. 2B,C): (1) ca. 20 cm thick mixture of coarse sand, fine gravel, and diamicton, which are possibly reworked; (2) 160 cm of uniform grey oxidized fine sand; (3) 70 cm of silt; and (4) 50 cm of coarse sand.

Three charcoal samples were collected from the fine sand unit, and one was collected from the coarse sand unit. The charcoal pieces range from 6.7 to 7.1 ka BP by radiocarbon dating at the Beta Analytic laboratory, USA (Table 1). Four samples were also collected for OSL dating at the OSL laboratory of the Institute of Mountain Hazards and Environment, Chinese Academy of Sciences (IMHE, CAS). The OSL dating results are shown in Table 2. Additionally, OSL dating of samples from each of the lower three units was performed on a Lexsyg Research automatic TL/OSL instrument at the IMHE, CAS. The sample from the silt unit gave an OSL age of 7.6 ± 0.7 ka, which is consistent with the radiocarbon age. The other two samples gave ages of 18.5 ± 1.7 ka (fine sand) and 15.3 ± 1.4 ka (coarse sand) (Fig. 2D); the difference may be due to their larger grain size, or lack of bleaching in a catastrophic flood event^20^. Combining the OSL and radiocarbon dating results gives a probable age of ~ 7 ka BP for the Shuangyu SWD.

**Table 1:** Radiocarbon dating results for samples from the Shuangyu slackwater deposit.

Sample	Material analysed	Percent modern carbon	Modern carbon fraction	Age (BP)
SC1	Charred material	41.73 ± 0.16	0.4173 ± 0.0016	7020 ± 30
SC2	Organic sediment	42.94 ± 0.16	0.4294 ± 0.0016	6790 ± 30
SC3	Charred material	43.10 ± 0.16	0.4310 ± 0.0016	6760 ± 30
SC4	Charred material	42.89 ± 0.16	0.4289 ± 0.0016	6800 ± 30

**Table 2:** Sample dose rate and Optically Stimulated Luminescence dating results for the Shuangyu slackwater deposit.

Sample	Depth (m)	K (%)	Th (10^–6^)	U (10^–6^)	Dosage rate (Gy/ka)	Number of test pieces	De/Gy	OSL age (ka)
SO1	2.3	3.07 ± 0.04	14.24 ± 0.70	2.44 ± 0.40	3.88 ± 0.28	8^a^ + 12^b^	71.6 ± 4.21	18.5 ± 1.7
SO2	–	–	–	–	–	–	–	–
SO3	3.4	3.22 ± 0.04	22.97 ± 0.80	1.74 ± 0.30	4.61 ± 0.34	8^a^ + 12^b^	35.1 ± 1.83	7.6 ± 0.7
SO4	3.9	2.36 ± 0.04	25.96 ± 0.80	1.46 ± 0.30	4.16 ± 0.30	8^a^ + 12^b^	63.8 ± 3.83	15.3 ± 1.4

^a^Number of test pieces using the Single Aliquot Regenerative-dose method.

^b^Number of test pieces using the Standardized Growth Curve method.

**Figure 3:**
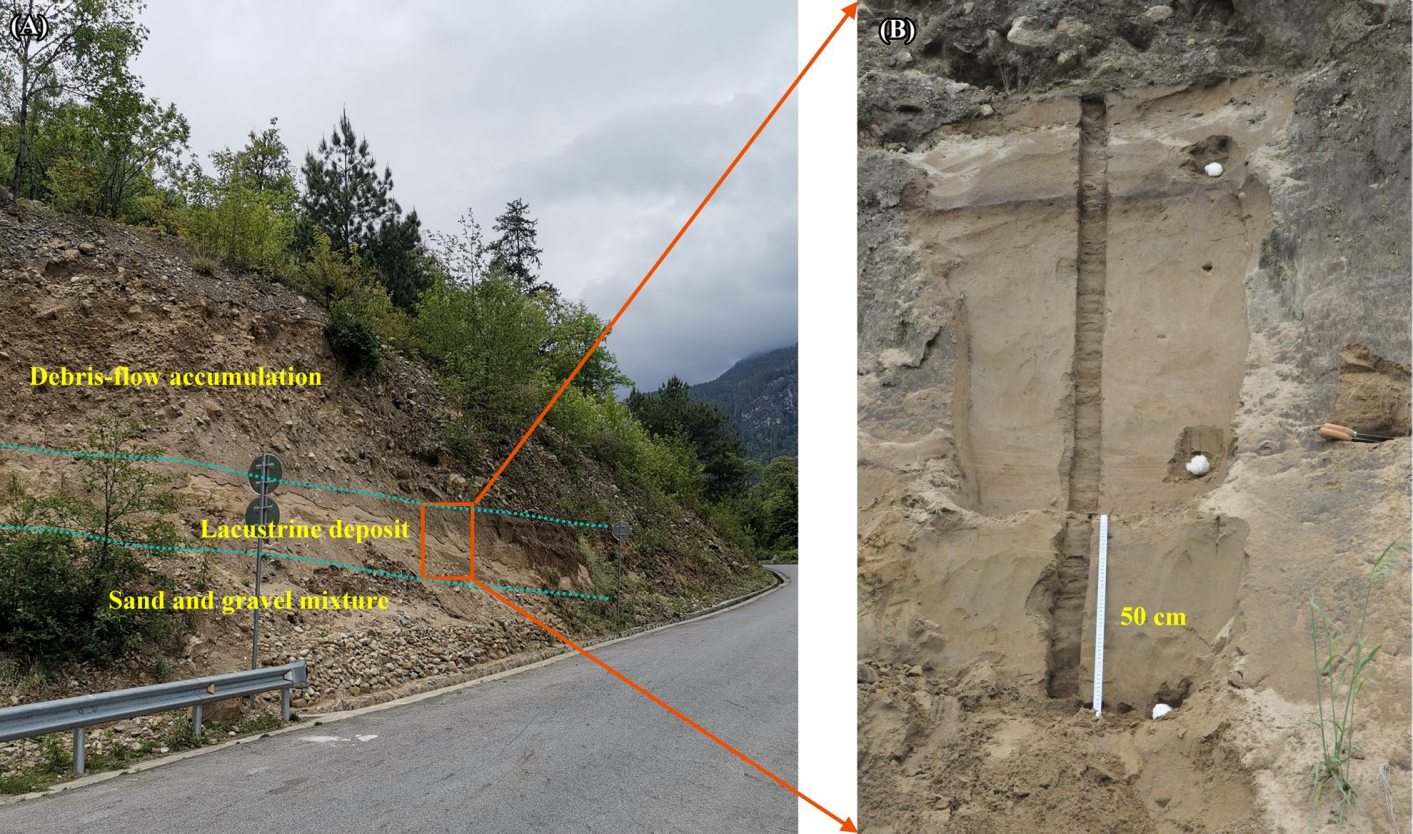
(**A**).The sediment sequence at the outlet of Dayi creek. (**B**) Section of the lacustrine deposit between the debris-flow accumulation and the mixed layer. The ruler is 50 cm long.

In addition, we found lacustrine deposits (N 30° 09′ 05″, E 94° 59′ 42″) at the mouth of Dayi Creek, a Yigong tributary, 2 km upstream of the Shuangyu SWDs (Fig. 1C). The 2.2 m parallel lacustrine lamination with yellow–brown silt is elevated ca. 40.0 m above the adjacent floodplain. It is capped by a 4.3 m debris-flow accumulation composed of angular and sub-angular gravels and boulders. A 1.5 m no-bedding mixture with sand and gravels is exposed underneath the lacustrine lamination (Fig. 3A). Two pieces of charcoal were taken from the silt deposit and radiocarbon dating was performed at the Beta Analytic laboratory, USA (Fig. 3B). The ^14^C ages are determined to be 6.3 ka BP and 6.6 ka BP, which means that the Dayi lacustrine deposits are a few centuries younger than the Shuangyu SWD. The sedimentary sequence is located at the mouth of the Dayi Creek branch on the right bank of the Yigong River. The bank is a concave bend behind a narrow reach. The transport capacity of the 7 ka BP superflood decreased and a large volume of sediment carried by the superflood stopped here. We speculate that the paleoflood deposits jammed the outlet of the Dayi, forming a small temporary dammed lake in the creek. Inflow silt was deposited in the dammed lake and produced lacustrine deposits. The upper debris-flow accumulation on the lacustrine deposits implies that large-scale debris flows probably occurred in Dayi and breached the temporary dam.

### The modern flood SWDs

**Figure 4:**
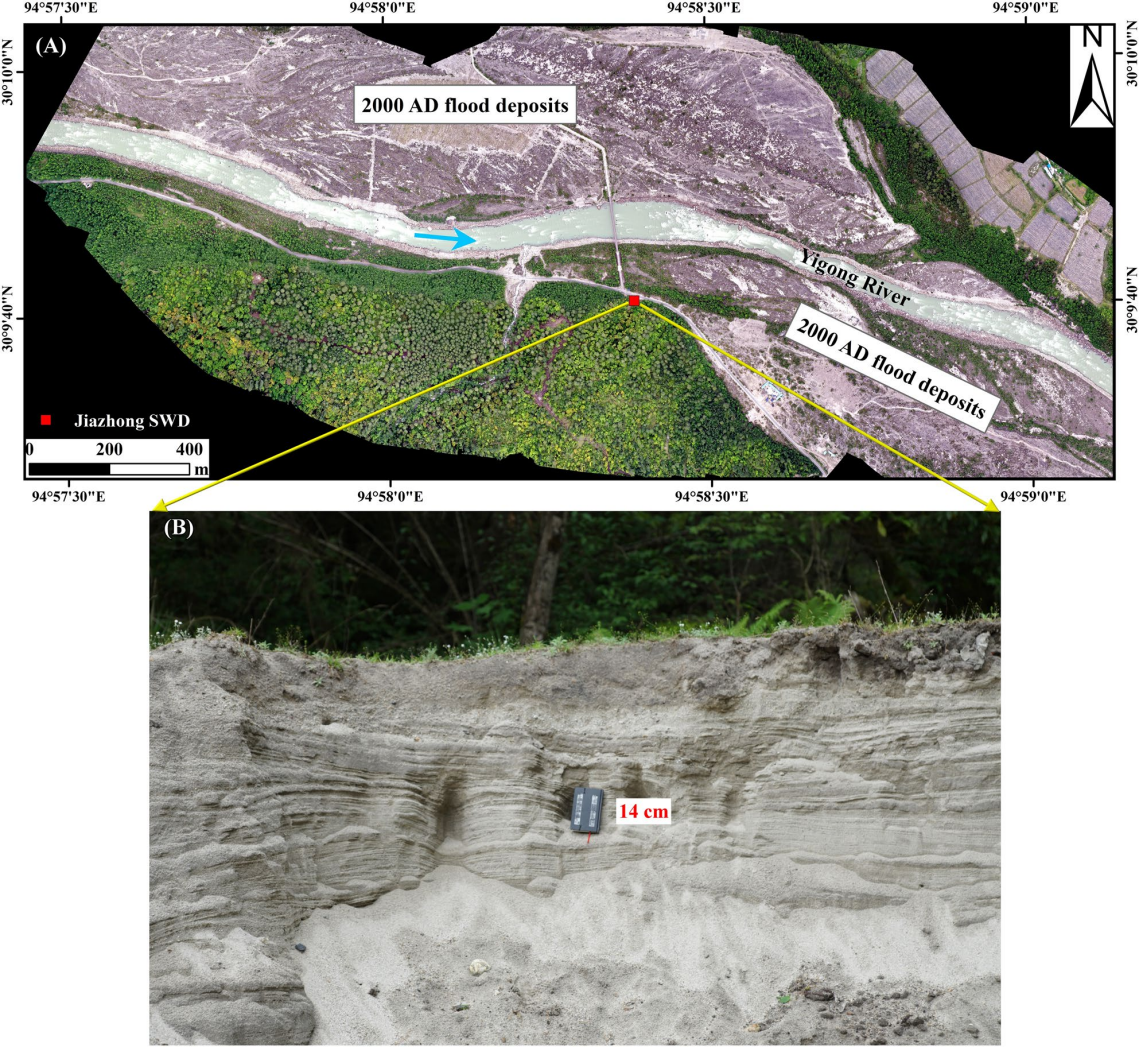
(**A**) Location of slackwater deposits (SWDs) on the opposite bank of Jiazhong Village. Orthographic image taken on May 11, 2020, by the Dajiang unmanned aerial vehicle (UAV). (**B**) Exposed sedimentary section of the SWDs. The notebook is 14 cm high.

Another SWD was found on the opposite bank of Jiazhong village, a hydraulically sheltered area 3.9 km downstream of the 2000 AD breached dam (Fig. 1C). The Jiazhong SWD is located within a cove on the right bank, and its surface is a grassland ~ 6.0 m higher than the floodplains on both banks (Fig. 4A). The excavated section is a 1.2 m thick sequence of fine to medium-grained sand capped by an ~ 10 cm thick sandy loam ~ 21.0 m above the river level. The dark grey fine sand is interbedded with three units of ~ 10 cm thick grey medium sand and mingled with sub-angular gravels (Fig. 4B). The 70 cm upper part is nearly parallel laminated, but the lower part inclines to the river at an angle of ~ 5°, indicating an original local gradient and flow direction. A piece of charcoal collected in the middle of the SWD section was dated at the Beta Analytic laboratory. The measured percent modern carbon is 100.12 ± 0.37 and the radiocarbon age ranges from 1880 to 1956 AD with 87.3% probability. Monsoon seasonal floods that peak at ~ 2100 m^3^/s are unlikely to reach the location of the Jiazhong SWD. The uppermost thin sandy loam obviously formed in a relatively short period. Moreover, the Jiazhong SWD is located in the edge of the 2000 AD inundation area (Fig. 1C) and its elevation is close to the 2000 AD flood deposits next to it (Fig. 4A). Therefore, we interpret the Jiazhong SWD as the product of the 2000 outburst flood. The Jiazhong SWD level can be used as the flood stage indicator of the 2000 event.

### Peak flow reconstruction

There are no direct records of the basic parameters of the 1902 AD and 7 ka BP floods such as breach depth and peak discharge. The well-documented 2000 AD flood can be used as a benchmark to aid the reconstruction of historic events. The peak 2000 AD discharge at Tongmai Bridge has been estimated as 126,400 m^3^/s^13^, 120,000 m^3^/s^12^, and 124,000 m^3^/s^21^, with a peak water depth of 52 m. We compare peak discharge calculations for the 2000 AD flood using 15 empirical formulas and known data on breach depth, dam height, volume of released water, and impounded water volume (Tables 3, 4)^22^. The MacDonald and Langridge-Monopolis (MLM)^23^ formula produced the closest estimate to the measured discharge, at ~ 130,000 m^3^/s. Therefore, the MLM formula is used to estimate the peak discharge of the 1902 AD event using a breach height of 75 m^12^ and the empirical relationship between the lake area and its volume from Delaney and Evans^15^ (Table 4). The resulting discharge of the 1902 AD event is ~ 182,000 m^3^/s, more than 50 times a normal seasonal flood discharge in the Yigong.

**Table 3:** Estimates of peak discharge for the outburst flood generated by the 2000 AD Yigong dam failure using 15 empirical models listed in Liu et al.^21^.

Author	Model*	Publication date	Peak discharge (m^3^/s)
Kirkpatrick	Q_p_ = 1.268(H_w_ + 0.3)^2.5^	1977	28,835
SCS	Q_p_ = 16.6H_w_^1.85^	1981	27,528
USBR	Q_p_ = 19.1H_w_^1.85^	1988	31,674
USBR	Q_p_ = 48H_w_^1.63^	1988	32,963
Hagen	Q_p_ = 0.54(V_s_-H_d_)^0.5^	1982	24,239
Singh and Snorrason	Q_p_ = 13.4H_d_^1.89^	1984	26,084
Singh and Snorrason	Q_p_ = 1.776V_s_^0.47^	1984	41,922
MacDonald and Langridge-Monopolis	Q_p_ = 3.85(H_w_V_w_)^0.41^	1984	129,944
Costa	Q_p_ = 1.122V_s_^0.57^		225,643
Costa	Q_p_ = 0.981(V_s_H_d_)^0.42^	1985	42,698
Costa	Q_p_ = 2.634(V_s_H_d_)^0.44^	1988	2,998,208
Evens	Q_p_ = 0.72V_w_^0.53^	1986	61,459
Froechlich	Q_p_ = 0.607H_w_^1.24^V_w_^0.295^	1995	48,528
Webby	Q_p_ = 0.0443g^0.5^V_w_^0.365^H_d_^1.4^	1996	94,314

*Where Q_p_ is the outburst flood peak discharge, V_w_ is the water released (m^3^), V_s_ is the barrier lake volume, H_w_ is the breach depth, and H_d_ is the dam height. In this study, we only considered complete dam breach; thus, V_w_ is the same as V_s_, and H_w_ is the same as H_d_.

**Table 4:** Basic parameters of the Yigong barrier lakes^15^ and estimated peak discharges of associated outburst floods in 1902 and 2000 AD.

Year	Breach depth (m)	Lake area (km^2^)	Lake volume^b^ (Gm^3^)	Peak discharge^c^ (m^3^/s)
2000	55	48.93	2.015	129,944
1902	75^a^	56.9	3.378	182,375

^a^It is assumed that the breach depth is equal to the minimum dam height given by Shang et al.^12^.

^b^Volume calculated using $V = A^{3.424} *3304.5$^15^, where A is the area of impounded water.

^c^Peak discharge calculated using $Q_{p} = 3.85*(H*V)^{0.41}$^23^, where H is the barrier lake water depth and V is the lake volume.

**Figure 5:**
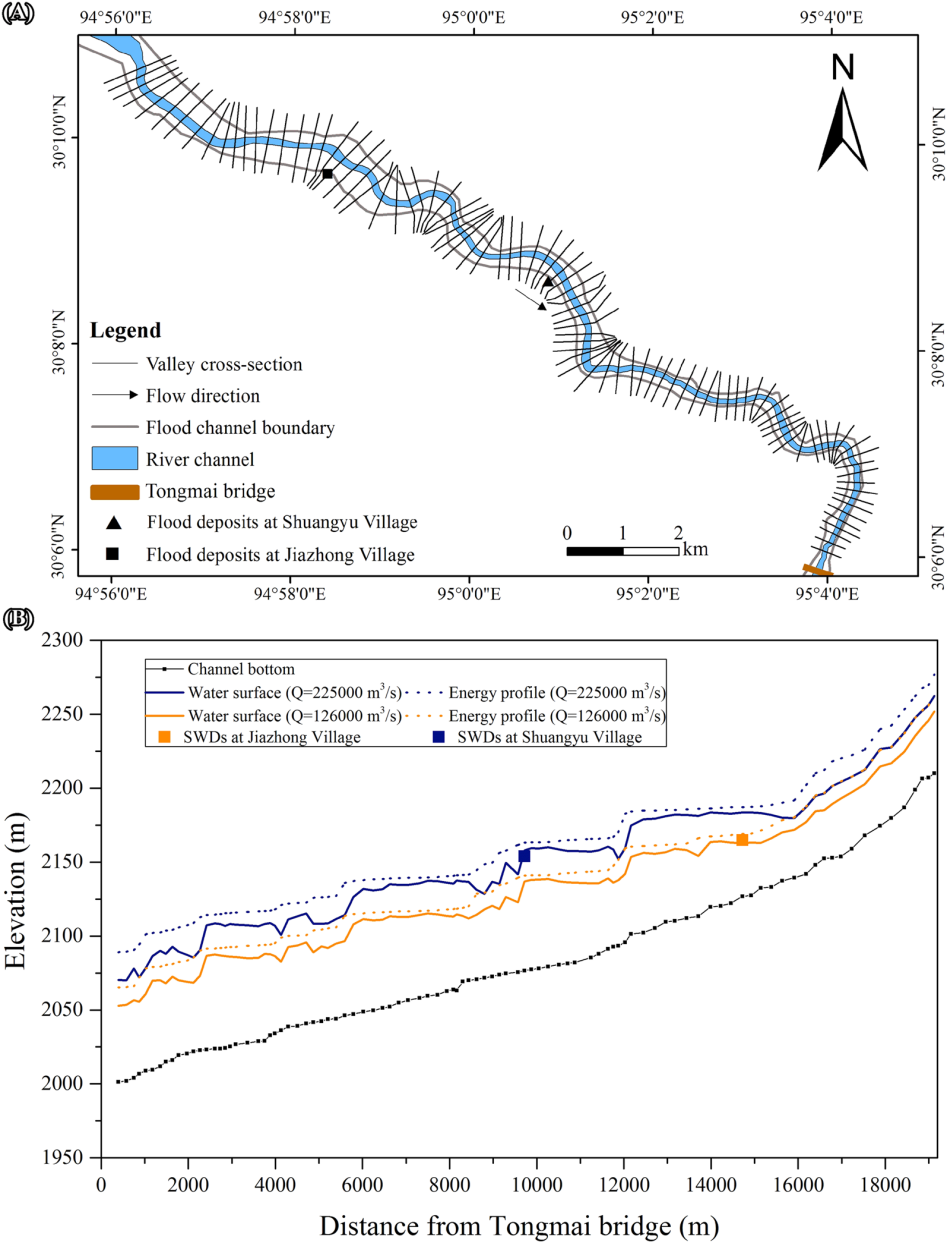
(**A**) The locations of 97 cross-sections used in the HEC-RAS mapper component of HEC-RAS 5.0.7 for the 1D step-backwater computation from the breached dam to the bridge. (**B**) Longitudinal profiles of the water surface and energy grade elevations calculated using the step-backwater method. Peak discharges of 126,000 m^3^/s and 225,000 m^3^/s provided the best approximation for the levels of the SWDs at Jiazhong and Shuangyu.

SWD surface elevation is widely used in paleoflood hydraulic reconstruction as a PSI^18,24^. The most commonly used paleoflood peak discharge estimation technique is the 1-D step-backwater method^18,25^. We applied the method to the 2000 AD and 7 ka BP events using 1-D steady flow analysis of HEC-RAS 5.0.7 software^26^, assuming a subcritical flow regime. Contour lines interpolated from the ALOS DEM (released in 2014 with 12.5 × 12.5 m spatial resolution) correspond well with channel shape and location in the ETM + image in 1999, so they provide an acceptable record of pre-2000 AD topography. The starting cross-section for the step-backwater calculation was set at the Tongmai Bridge bedrock section. A total of 97 cross sections were extracted along the 19.2 km reach with an average spacing of 200 m using the RAS Mapper of HEC-RAS 5.0.7 (Fig. 5A).

Expansion and contraction coefficients of 0.1 and 0.3 were specified based on channel width^26^. Manning’s roughness coefficient *n* was calibrated using the peak discharge (126,000 m^3^/s) and water depth (52 m) of the 2000 AD flood. The computed water surface elevation at the Tongmai and Jiazhong cross-sections corresponded well with the 2000 AD flood level when Manning’s roughness coefficient was set as 0.03 and 0.035 for the river channel and floodplain, respectively. The roughness values are also the suggested values for the mountain stream such as the Yigong River (channel bottom with gravels, cobbles, and few boulders, no vegetation in channel, and steep banks with trees and brush probably submerged)^26^. The calculated values of the velocity and flow area of the 2000AD flood at Tongmai Bridge are 15.7 m/s and 8350 m^2^, respectively, which are close to those estimated by Delaney and Evans^15^. Sensitivity tests in the model show that a 25% variation in Manning’s roughness coefficient results in variation of 2.65–2.76% in flood water depth and variation of 0.12–0.15% in peak discharge. Using these values of the coefficients, a discharge of 225,000 m^3^/s provides the best approximation for the Shuangyu deposits (Fig. 5B). For the 7 ka BP flood, the velocity and the flow area at Tongmai Bridge are 19.5 m/s and 12,360 m^2^, respectively. The paleoflood with such a magnitude can yield a bed shear stress of 5 kPa and move large boulders up to 5–6 m in diameter^8^.

## Discussions

**Figure 6:**
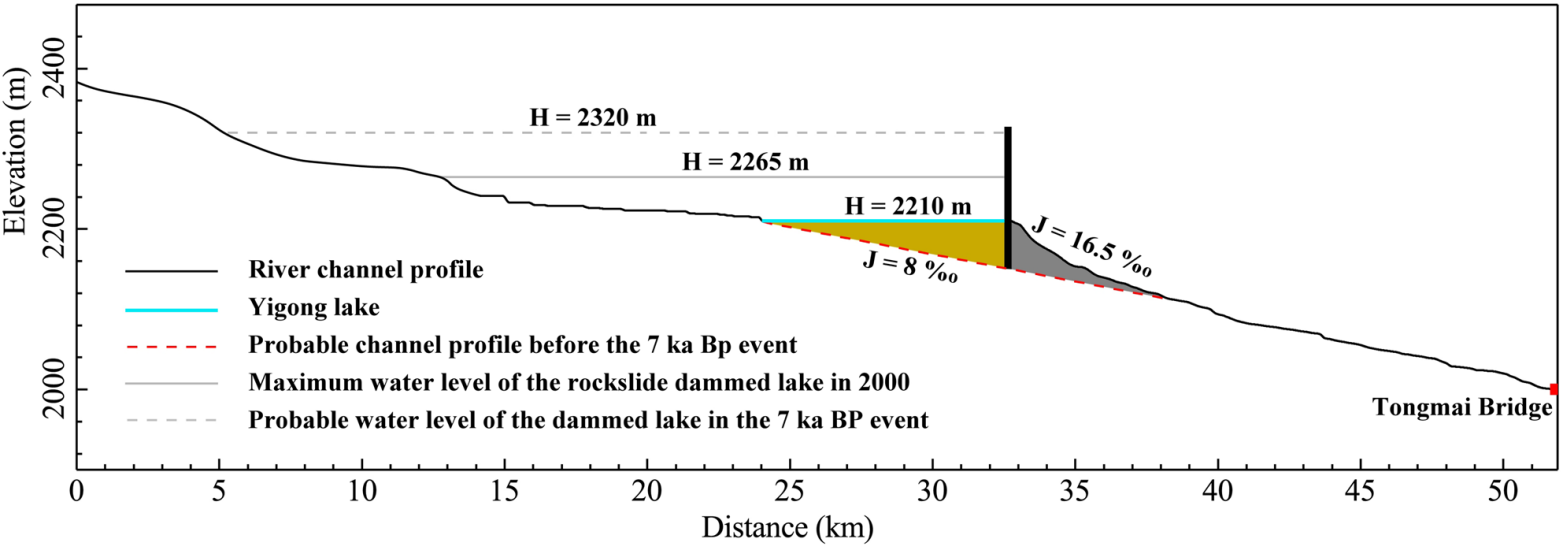
Profile of the Yigong River downstream and probable water level of the 7 ka BP dammed lake according to a peak flow of 225,000 m^3^/s.

The landslide dams and outburst floods changed the longitudinal profile of the Yigong River dramatically. Delaney and Evans^15^ plotted a speculative pre-existing profile derived from SRTM-3 data. They proposed that it could be the river valley profile before the 1900 event (the 1902 event in this paper). The intersection point of the pre-existing channel with the present channel is ~ 5.8 km downstream of the breached dam (Fig. 6). The channel slope from the dam to the intersection point is 16.5‰, which is double the pre-channel slope. This reach was filled with quick deposits of landslides and outburst floods. The sediment supply from the landslides keeps the bed stable under such a steep slope. From the intersection point to the Tongmai Bridge, the channel slope is 8‰, which is probably a natural slope without the disturbance of landslide damming. The paleo-channel elevation a.s.l. is ~ 2150 m at the dam (Fig. 6).

**Figure 7:**
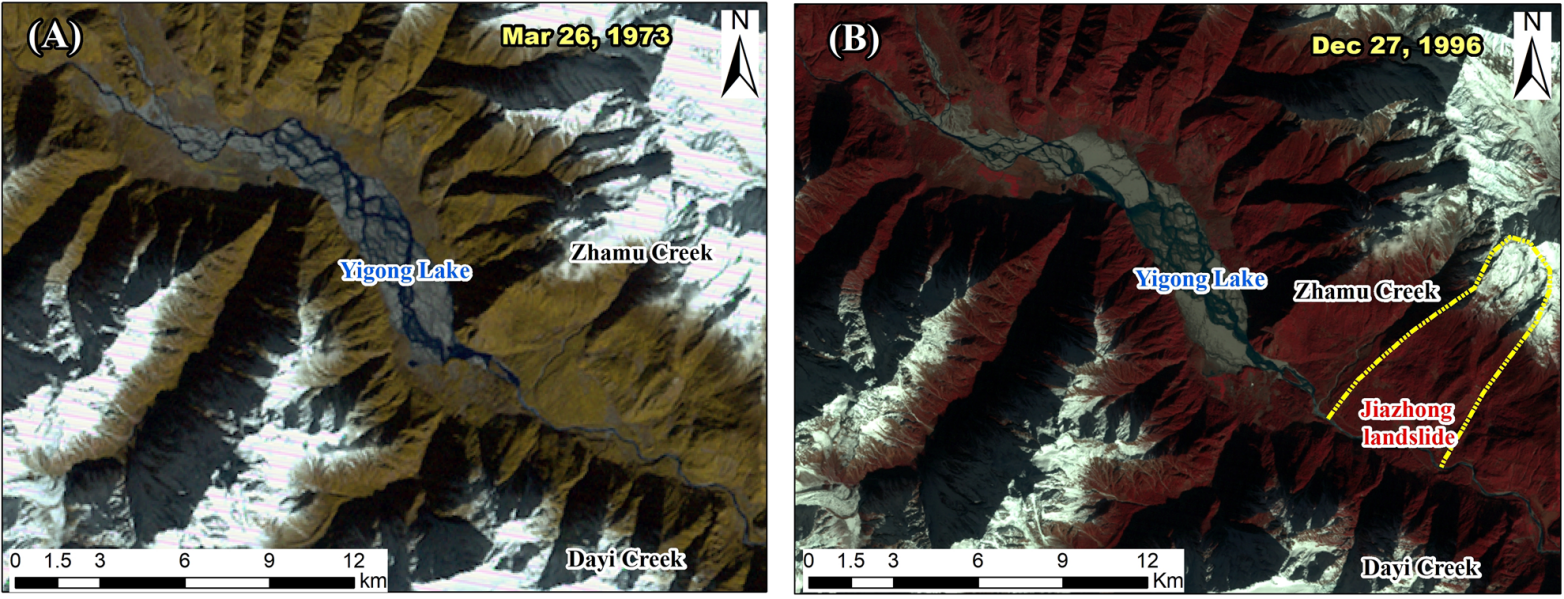
(**A**) KeyHole-9 satellite image of Yigong Lake on March 26, 1973, and (**B**) TM satellite image of Yigong Lake on December 27, 1996. The yellow outline marks the ancient landslide at Jiazhong village. The maps were created using a licensed ArcGIS 9.3 software (https://support.esri.com/en/downloads).

The lake and braided stream system completely developed as early as 1973 according to the KeyHole-9 satellite image (Fig. 7A). The lake level and fluvial deposits show little change even after 23 years (Fig. 7B). We estimate that the trapped sediment in the lake is ~ 0.26 billion m^3^ with an average lake width of 1 km. Considering the low sediment concentration of the Yigong River, it should take more than a thousand-year span to trap such a large volume of sediment. It is reasonable to conclude that Yigong Lake likely formed as early as 7 ka BP and that the 1902 landslide accumulation overlapped with the previous residual dam. Assuming the paleo-dam breached to an elevation of 2210 m a.s.l., it is estimated that the released water volume required for producing the peak flow of 225,000 m^3^/s was 6.88 Gm^3^. This means that the water level was 2320 m in elevation when the 7 ka BP flood occurred, and the height of the dam was at least 170 m (Fig. 6). The stable knickpoint induces backwater aggradation and protects the upstream river channel from incision. We conjecture that the 7 ka BP dam may have been caused by an ancient landslide at Jiazhong village or debris flows in Zhamu or Bailong catchments from the remaining site-specific alluvial fan or landslide terrace (Fig. 7B).

Glacial and landslide dams in Tibetan-Himalayan rivers contribute greatly to the long-term stability of river knickpoints in the Himalayan syntaxes and hence impede incision into the interior of the Tibetan Plateau^3,4^. Previous research suggests that landslide damming is spatially random and that the river incision is inhibited mainly by moraine dams formed at the same locations during glacial advances^4,27^. However, the Yigong cases and recent large-scale landslides triggered by earthquakes or ice-rock avalanches at the entrance of Tsangpo Gorge and on the Parlung River demonstrate that some landslide dams recur at the same location and repeatedly block the rivers near the eastern syntaxis of the Himalaya^28,29^. Correspondingly, this implies that recurrent landslide dammings may play a primary role in the stability of some knickpoints when glacial or moraine dams cannot reach the river trunk during the deglaciation period.

## Conclusions

Dammed lake outburst floods are common in the margin of the Tibetan Plateau. In this paper, two slack water deposits and one lacustrine deposit near the 1902 and 2000 AD Yigong landslide dams are reported in detail. From these sediment records, a paleoflood event of ~ 7 ka BP is identified by radiocarbon and OSL dating tests. The 1-D backwater stepwise method gives a 225,000 m^3^/s peak flow for the paleoflood. The peak discharge of the 1902 AD flood is ~ 182,000 m^3^/s by using the best fit empirical model that is calibrated with the 2000 AD flood. These dams and associated outburst floods strongly influenced the river landscape via altering the channel longitudinal profile downstream of the Yigong lake. It is estimated that a volume of ~ 0.26 billion m^3^ of sediment or even more has been aggraded upstream of the 2000 AD dam. Large volume of sediments carried by the dam-break outburst floods rapidly settled and covered on the paleo-channel right behind the dams, leading to an increase in the channel slope from 8‰ to 16.5‰. Moreover, at least three superfloods > 10^5^ m^3^/s have occurred on the Yigong since 7 ka BP, indicating that the recurrence interval of Holocene outburst floods on the southeastern margin of the Tibetan Plateau is much shorter than that of monsoon floods with the same magnitude. The dominant effect of outburst floods should be accounted for in long-term landscape evolution models of the southeastern margin.

## Data Availability

All data and material are available in the main text or in cited resources mentioned in the text.

## Code availability

All software applications used in this paper are free or open-source and can be found in cited resources mentioned in the text.
